# Increased complications of proximal femur fractures during the COVID-19 pandemic: a nationwide medical claims database study in Japan

**DOI:** 10.1007/s00774-025-01611-0

**Published:** 2025-06-10

**Authors:** Hidetatsu Tanaka, Kunio Tarasawa, Yu Mori, Kiyohide Fushimi, Kenji Fujimori, Toshimi Aizawa

**Affiliations:** 1 Department of Orthopaedic Surgery, Tohoku University Graduate School of Medicine, 1-1 Seiryo-Machi, Aoba-Ku, Sendai, Miyagi 980-8574 Japan; 2 Department of Health Administration and Policy, Tohoku University Graduate School of Medicine, 2-1 Seiryo-Machi, Aoba-Ku, Sendai, Miyagi 980-8574 Japan; 3 Department of Health Policy and Informatics, Tokyo Medical and Dental University Graduate School of Medicine and Dental Sciences, 1-5-45 Yushima, Bunkyo-Ku, Tokyo, 113-8519 Japan

**Keywords:** COVID-19, Proximal femur fracture, Nationwide cohort study, Complication, Mortality

## Abstract

**Introduction:**

Coronavirus disease 2019 (COVID-19) is a worldwide pandemic, and mortality increases in elderly patients with comorbidities. This study aims to examine the in-hospital complications and mortality for elderly patients with proximal femur fractures during the COVID-19 pandemic or countermeasure periods.

**Materials and methods:**

The proximal femur fractures undergoing surgery during the COVID-19 pandemic or countermeasure period were compared with the pre-pandemic in a Japanese national inpatient data. The assessed outcomes were the development of pneumonia, deep vein thrombosis (DVT), pulmonary embolism (PE), and mortality during hospitalization in two periods, and those for COVID-19-positive patients.

**Results:**

A total of 284,922 proximal femur fractures aged over 65 years were included. In the COVID-19 pandemic period compared to the pre-pandemic, the odds of pneumonia, DVT, PE, and mortality decreased to 0.942 (95% confidence interval [CI]: 0.901 − 0.986, *P* = 0.0102) and 0.839 (95% CI: 0.745 − 0.946, *P* = 0.004), increased to 1.153 (95% CI: 1.112 − 1.195, *P* < 0.001), and 1.048 (95% CI: 0.982 − 1.118, *P* = 0.1554), respectively. For COVID-19 positivity at admission, the odds of PE increased significantly to 12.95 (95% CI: 8.795 − 19.06, *P* < 0.001). For COVID-19 positivity during hospitalization, the odds of pneumonia and mortality were increased to 2.896 (95% CI: 1.820 − 4.608, *P* < 0.001) and 6.303 (95% CI: 3.440 − 11.55, *P* < 0.001), respectively.

**Conclusion:**

These findings alert healthcare professionals and patients to the elevated complications, especially PE rate for proximal femoral fracture with COVID-19 positive.

**Supplementary Information:**

The online version contains supplementary material available at 10.1007/s00774-025-01611-0.

## Introduction

Since late 2019, the world has faced the global pandemic of coronavirus disease 2019 (COVID-19), and the World Health Organization declared it as a global pandemic in March 2020 [1–4]. Hospitalization, severe illness, and mortality increase among patients over the age of 65 and with comorbidities such as hypertension, diabetes, cardiovascular disease, chronic respiratory disease, compromised immune status, cancer, and obesity [5, 6]. Over the country, there were irregular waves of nationwide epidemics, with the first case of COVID-19 in February 2020 in Japan [7]. To prevent COVID-19 from spreading, four times of emergency status and two times of semi-emergency status were declared in Japan from April 2020 to March 2022.

Proximal femur fracture among older adults often results in decreased independence and quality of life and increased mortality [8]. Early surgery is recommended for proximal femoral fractures [9–11], because surgical delay is associated with worse outcomes [9, 12, 13]. Although concerns that older patients with hip fractures may be particularly susceptible to developing severe COVID-19 infection in the perioperative period, fragility fractures, including proximal femur fracture, have not decreased during the COVID-19 pandemic under social distancing and travel restrictions as a society [14–20]. Patients with a proximal femur fracture who tested positive for COVID-19 had a higher mortality rate compared with those who tested negative [21–23]. During the COVID-19 pandemic or countermeasure periods, there had been a need for early surgery with COVID-19 prevention measures such as clinical judgment, appropriate precautions, and universal testing.

Research on the impact of the COVID-19 pandemic on hip fracture surgery, postoperative complications, and mortality during hospitalization is limited compared to the period before the pandemic. We delineated two periods for our analysis: the pre-COVID-19 pandemic and the response period, each spanning 2 years. The primary aim of this study is to elucidate the impact of the COVID-19 pandemic on elderly patients with proximal femur fractures by comparing outcomes from these two timeframes. Additionally, the secondary objective seeks to assess the hospitalization outcomes and determine if the prognosis for patients with COVID-19-positive proximal femoral fractures differs from those who tested negative.

## Materials and methods

This study used data from the Japanese Diagnosis Procedure Combination (DPC) database with the ethical standards of the Declaration of Helsinki and approved by the Tokyo Medical and Dental University (approval No. M2000-788) and Tohoku University Graduate School of Medicine (approval No. 2021–1-1082). The study was performed in accordance with the Strengthening the Reporting of Observational Studies in Epidemiology (STROBE) reporting guideline [24].

## Data source

The DPC data in this study included approximately 1100 hospitals, covering around 70% of all hospitalization episodes annually in Japan, reflected in the country's clinical practices. The anonymized data contains the hospital identification number, patient age, gender, diagnosis (coded according to the International Classification of Diseases, Tenth Revision codes (ICD-10) [25], dates of hospital admission and discharge, discharge status, drugs and procedures, including surgery. In addition, diagnoses of admissions, pre-existing comorbidities at admissions, and post-admission complications during hospitalization are separately recorded.

## Study design

**Fig. 1:**
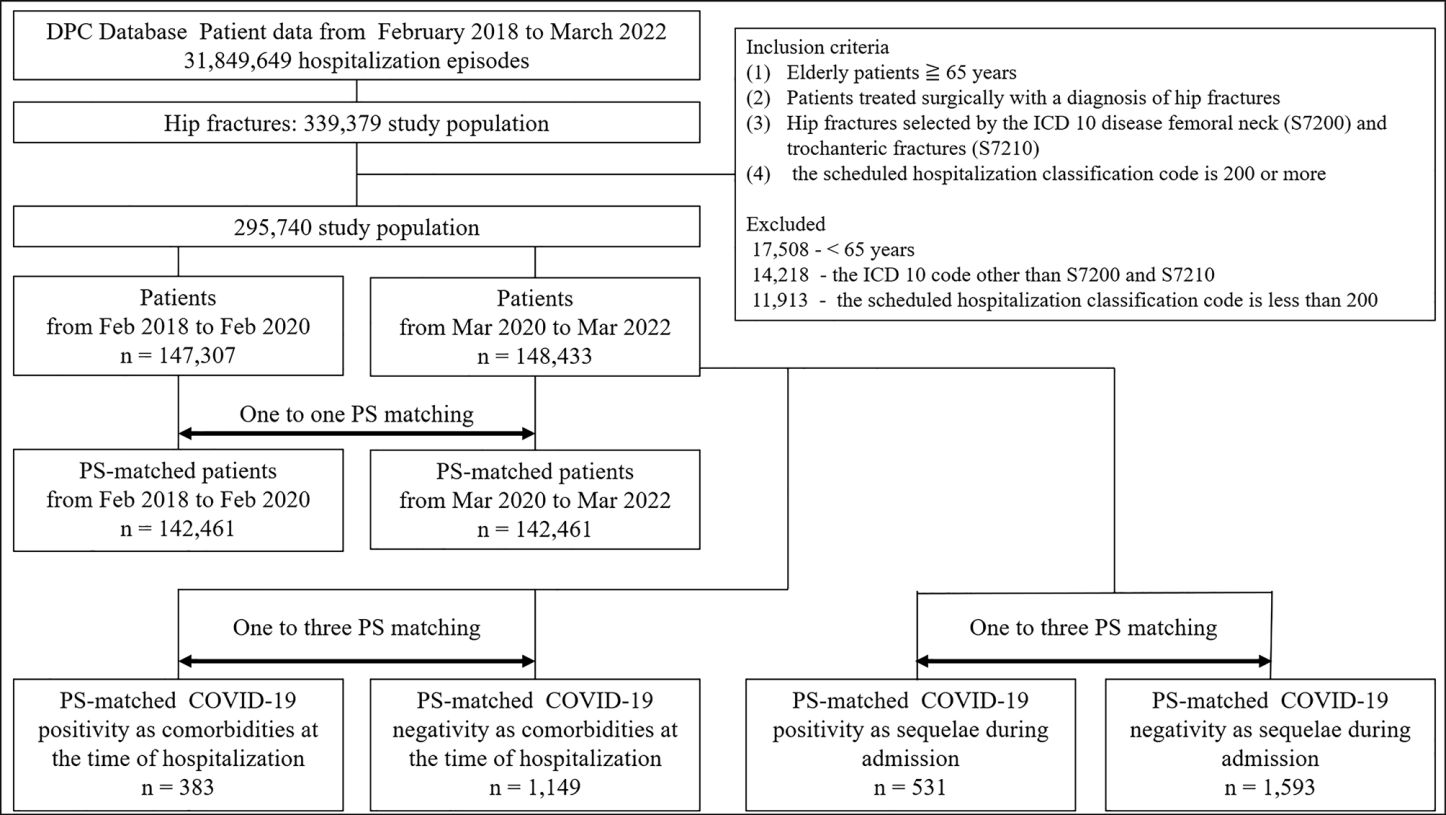
Flow diagram of patient selection for elderly hip fracture and PS matching

The Japanese National Administrative DPC reimbursement system database was retrospectively reviewed. From January 2018 to March 2022, surgical treatment was performed on a database of 339,379 proximal femur fracture patients. The clinical study focused on proximal femur fractures in older adult patients during the COVID-19 pandemic or countermeasure periods and pre-COVID-19 pandemic. Based on the ICD-10, two proximal femur fractures were selected: femoral neck (S7200) and trochanteric (S7210) fractures. The inclusion criteria for the cohort were set to (1) the DPC code was S7200 and S7210 with diseases requiring the most medical resources, (2) aged 65 years and over, and (3) the scheduled hospitalization classification code is 200 or more, meaning unscheduled (emergency) hospitalization. Subtrochanteric fractures (S7220) were excluded from the study because of different pathogenesis. To assess the influence of COVID-19 in Japan since March 2020, the study period was divided into two distinct phases: February 2018 to February 2020, representing the pre-COVID-19 pandemic period, and March 2020 to March 2022, representing the COVID-19 pandemic or countermeasure period. Additionally, to examine the impact of COVID-19, we analyzed the patients with COVID-19 positivity as comorbidities at the time of hospitalization and the patients with COVID-19 positivity during hospitalization from March 2020 to March 2022. COVID-19 positivity was identified using ICD-10 codes of U07.1 recorded at the time of admission or during hospitalization. A flow diagram of the study design is shown in Fig. 1.

## Data selection

One-to-one Propensity Score (PS) matching was employed, comparing individuals from February 2018 to February 2020 with those from March 2020 to March 2022. Additionally, one-to-three PS matching was performed on whether the positivity of COVID-19 during hospitalization or not, and positivity of COVID-19 during hospitalization from March 2020 to March 2022. The covariates utilized for a confounding setting included age, gender, and comorbidities, including hypertension, dementia, ischemic heart disease, cerebrovascular disease, chronic renal dysfunction, chronic lung disease, and diabetes. Comorbidities were defined based on ICD-10 codes (Table S1). C-statistics were used to assess the discriminative power of the models. PS estimates were used to perform nearest-neighbor matching without replacement, with the PS estimates being used as the calipers; the caliper was set to 0.2 times the standard deviation of the PS estimate. This resulted in matched pairs and the establishment of PS-matched control and treatment groups.

## Outcomes

Assessed outcomes included the development of hospital-acquired pneumonia, deep vein thrombosis (DVT), pulmonary embolism (PE), and mortality during hospitalization. Pneumonia, DVT, and PE were defined based on ICD-10 codes (Table S1). Mortality was defined from discharge outcome codes 6 and 7. The pneumonia was defined as any pneumonia occurring after the surgery, including both bacterial and viral causes. Furthermore, waiting days before surgery, general anesthesia rate, and the intensive care unit (ICU) management have been investigated.

## Statistical analyses

All data are expressed as mean ± standard deviation. Significant differences between the two groups were examined using the *χ*2 test and Student's *t* test for each parameter. Shapiro–Wilk test was used to evaluate the normally distributed variables. Univariate logistic regression analysis has been used to assess the association between hospitalization on the site from March 2020 to March 2022 and the occurrence of pneumonia, DVT, PE, and mortality during hospitalization. Following the initial variate analyses, a multivariate logistic regression analysis was performed. Univariate logistic regression analysis has also been used to investigate the association between positivity of COVID-19 at the time of hospitalization or during hospitalization on the site from March 2020 to March 2022, and the occurrence of pneumonia, DVT, PE, and mortality during hospitalization. All statistical tests were two-tailed; *p* values < 0.001 were considered significant. All analyses were performed using JMP version 17.2 (SAS, Cary, NC, USA). Due to the large sample size and the observational design of the study, a stricter significance threshold of *P* < 0.001 was used to account for the increased risk of Type I error and to avoid overinterpretation of marginal findings.

## Results

### Participants

295,740 patients meet the inclusion and exclusion criteria. After one-to-one PS matching, there were 142,461 patients each from February 2018 to February 2020 and from March 2020 to March 2022. Baseline demographic data, including age, gender, comorbidities, anticoagulant treatment, and osteoporosis treatment status in two periods, are shown in Table 1. The C statistic was 0.7253. Standardized mean differences (SMD) were < 0.1 for all parameters. The waiting days before surgery were significantly shorter during the COVID-19 pandemic or countermeasure periods. The general anesthesia rates were significantly higher during the COVID-19 pandemic or countermeasure periods. The anticoagulant medication rates had no significant difference. The osteoporosis treatment rate is higher during the COVID-19 pandemic or countermeasure periods. An active form of vitamin D and weekly bisphosphonates were mainly used for treatment (Table 1).

**Table 1:** Characteristics of patients before and after propensity score matching

	Feb 2018–Feb 2020	Mar 2020–Mar 2022	SMD
*n*	142,461	142,461	
Age	84.7 ± 7.6	84.7 ± 7.7	0.0008
Gender			
Men	30,713 (21.6)	30,742 (21.6)	0.0005
Women	111,748 (78.4)	111,719 (78.4)	
Comorbidities			
Hypertension	54,650 (38.4)	54,575 (38.3)	0.0011
Dementia	32,634 (22.9)	32,701 (23.0)	0.0011
Diabetes	26,422 (18.6)	26,351 (18.5)	0.0013
Cerebrovascular disease	14,039 (9.9)	13,983 (9.8)	0.0013
Ischemic heart disease	10,888 (7.6)	10,815 (7.6)	0.0019
Chronic renal dysfunction	8332 (5.9)	8179 (5.7)	0.0046
Chronic lung disease	2066 (1.5)	1970 (1.4)	0.0057
			p value
Waiting days before surgery	3.47 ± 3.60	3.17 ± 3.26	< 0.001※
General Anesthesia	86,503 (60.7)	89,603 (62.9)	< 0.001
ICU admission	5985 (4.2)	6304 (4.4)	0.003
Anticoagulant treatment	70,157 (49.3)	70,913 (49.8)	0.005
Edoxaban	38,188 (26.8)	41,077 (28.8)	< 0.001
Fondaparinux	1759 (1.2)	1515 (1.1)	< 0.001
Enoxaparin	5109 (3.6)	3388 (2.4)	< 0.001
Apixaban	4166 (2.9)	4698 (3.3)	< 0.001
Clopidogrel	7642 (5.4)	8145 (5.7)	< 0.001
Aspirin	17,307 (12.2)	16,290 (11.4)	< 0.001
Warfarin	5174 (3.6)	3954 (2.8)	< 0.001
Osteoporosis treatment	38,561 (27.1)	40,367 (28.3)	< 0.001
Bisphosphonates	15,157 (10.6)	17,033 (12.0)	< 0.001
Teriparatide	2377 (1.7)	1937 (1.5)	< 0.001
Denosumab	540 (0.4)	599 (0.4)	0.085
Active form of Vitamin D	27,861 (19.6)	29,953 (21.0)	< 0.001
SERM	2091 (1.5)	1867 (1.3)	< 0.001

Age and waiting days before surgery are shown as mean ± standard deviation; Data showed No. (%) with data except for Age and waiting days

*P* values of < 0.001 are considered significant by the Student’s test and *χ*2 test

SMD means standard mean difference; ICU means intensive care unit; SERM means selective estrogen receptor modulator

※ Student *t* test

### Assessment for risk factors for complications and mortality during hospitalization in COVID-19 pandemic or countermeasure periods

The associations between the COVID-19 pandemic or countermeasure periods and the development of pneumonia, DVT, PE, and mortality during hospitalization are analyzed. In the COVID-19 epidemic or countermeasure period compared to the pre-pandemic, the risk of developing pneumonia, DVT, PE, and mortality during hospitalization decreased to 0.942 (95% Confidence Interval [CI]: 0.901 − 0.986) and 0.839 (95% CI: 0.745 − 0.946), increased to 1.153 (95% CI: 1.112 − 1.195), and 1.048 (95% CI: 0.982 − 1.118), respectively. Significant associations were found in PE development (Table 2).

**Table 2:** Univariate logistic regression analysis of risk factors for hospital-acquired pneumonia, DVT, PE, and mortality during hospitalization

Factors	Total (*n*)	OR (95% CI)	*χ*2 statics	*p* value
Hospital-acquired pneumonia	8097	0.942 (0.901–0.986)	6.602	0.0102
DVT	1092	0.839 (0.745–0.946)	8.284	0.0040
PE	12,445	1.153 (1.112–1.195)	59.86	< 0.001
Mortality during hospitalization	3898	1.048 (0.982–1.118)	2.018	0.1554

DVT means deep vein thrombosis; PE means pulmonary embolism; OR means Odds Ratio; CI means confidence interval

*P* values of < 0.001 are considered significant by the *χ*2 test

Multivariable regression analysis of PE and mortality during hospitalization was performed for several variables including age, gender, waiting days before surgery, COVID-19 positivity at the time of hospitalization and during hospitalization, general anesthesia, and ICU admission (Table 3). For PE, an increase in waiting days for surgery by one day: 1.034 (95% CI: 1.030 − 1.038); COVID-19 comorbidities at the time of admission: 9.590 (95% CI: 7.708 − 11.93); ICU admission: 1.157 (95% CI: 1.064 − 1.258) were significantly elevated the risk. Conversely, increase in age by one year with a ratio of 0.996 (95% CI: 0.994 − 0.998); male sex: 0.685 (95% CI: 0.652 − 0.719); general anesthesia: 0.910 (95% CI: 0.877 − 0.944) was a substantial decrease in the risk of PE. COVID-19 positivity during admission was not a significant factor for PE. For mortality during hospitalization, an increase in age by one year with a ratio of 1.073 (95% CI: 1.068 − 1.079); male gender: 2.872 (95% CI: 2.687 − 3.069); an increase in waiting days for surgery by one day: 1.039 (95% CI: 1.033 − 1.045); COVID-19 positivity during hospitalization: 4.650 (95% CI: 3.307 − 6.538); and ICU admission 3.089 (95% CI: 2.803 − 3.403). COVID-19 comorbidities at the time of admission were not significant factors for mortality during hospitalization.

**Table 3:** Multivariate logistic regression analysis of risk factors for hospital-acquired pneumonia, DVT, PE, and mortality during hospitalization

Factors	PE			Mortality during hospitalization		
	OR (95% CI)	*χ*2 statics	*p* value	OR (95% CI)	*χ*2 statics	*p* value
Age	0.996 (0.994–0.998)	11.45	< 0.001	1.073 (1.068–1.079)	927.1	< 0.001
Gender (Male)	0.685 (0.652–0.719)	247.5	< 0.001	2.872 (2.687–3.069)	881.7	< 0.001
Waiting days before surgery	1.034 (1.030–1.038)	230.9	< 0.001	1.039 (1.033–1.045)	134.9	< 0.001
COVID-19 positivity at time of admission	9.590 (7.708–11.93)	285.7	< 0.001	0.467 (0.149–1.459)	2.227	0.1356
COVID-19 positivity during hospitalization	0.579 (0.348–0.966)	5.125	0.0228	4.650 (3.307–6.538)	54.06	< 0.001
General Anesthesia	0.910 (0.877–0.944)	24.76	< 0.001	0.904 (0.846–0.965)	9.06	0.0026
ICU admission	1.157 (1.064–1.258)	11.16	< 0.001	3.089 (2.803–3.403)	408.4	< 0.001

PE means pulmonary embolism; ICU means intensive care unit; OR means Odds Ratio; CI means confidence interval

*P* values of < 0.001 are considered significant by the *χ*2 test

### Impact of COVID-19 positivity at the time of admission for patients with proximal femur fractures

There were 383 and 1149 patients with COVID-19-positive comorbidities at the time of hospitalization after one-to-three PS matching according to age, sex, and comorbidities. The characteristics are shown in Table 4. SMD was < 0.1 for all parameters. For a COVID-19-positive patient, the waiting days before surgery were significantly longer. In COVID-19-positive patients, the rate of general anesthesia was less, and the rate of ICU admission was significantly higher. The associations between COVID-19 positivity at the time of hospitalization and the development of pneumonia, DVT, PE, and mortality during hospitalization are shown in Table 5. The PE development risk increased significantly to 12.95 (95% CI: 8.795 − 19.06).

**Table 4:** Characteristics of patients after propensity score matching for COVID-19 positivity as comorbidities at the time of hospitalization and during hospitalization from March 2020 to March 2022

	COVID-19 as comorbidities at the time of hospitalization			COVID-19 positivity during hospitalization		
	Positive	Negative	SMD	Positive	Negative	SMD
*n*	383	1149		531	1593	
Age	85.6 ± 7.6	85.6 ± 7.5	0.0020	84.8 ± 7.6	85.0 ± 7.6	0.0314
Gender						
Men	71 (18.5)	183 (15.9)	0.0692	139 (26.2)	373 (23.4)	0.0640
Women	312 (81.5)	966 (84.1)		392 (73.8)	1220 (76.6)	
Comorbidities						
Hypertension	200 (52.2)	578 (50.3)	0.0383	241 (45.4)	720 (45.2)	0.0038
Dementia	85 (22.2)	265 (23.1)	0.0208	145 (27.3)	446 (28.0)	0.0154
Diabetes	81 (21.2)	250 (21.8)	0.0148	118 (22.2)	373 (23.4)	0.0284
Cerebrovascular disease	41 (10.7)	125 (10.9)	0.0060	52 (9.8)	162 (10.2)	0.0126
Ischemic heart disease	26 (6.8)	103 (9.0)	0.0808	37 (7.0)	123 (7.7)	0.0289
Chronic renal dysfunction	12 (3.1)	39 (3.4)	0.0147	40 (7.5)	103 (6.5)	0.0418
Chronic lung disease	9 (2.4)	35 (3.1)	0.0430	12 (2.3)	41 (2.6)	0.0204
			*p* value			*p* value
Waiting days before surgery	4.17 ± 4.67	3.33 ± 3.42	< 0.001※	4.57 ± 8.19	3.24 ± 3.32	< 0.001※
General Anesthesia	202 (52.7)	924 (80.4)	< 0.001	314 (59.1)	1167 (73.3)	< 0.001
ICU admission	40 (10.4)	20 (1.7)	< 0.001	52 (9.8)	18 (1.1)	< 0.001

Age and waiting days before surgery are shown as mean ± standard deviation; Data showed No. (%) with data except for Age and waiting days

*P* values of < 0.001 are considered significant by the Student’s t test and *χ*2 test

SMD means standard mean difference; ICU means intensive care unit

※ Student’s *t* test

**Table 5:** Association between the occurrence of complications and COVID-19 positivity as comorbidities at the time of hospitalization and during hospitalization

	COVID-19 positivity at the time of hospitalization				COVID-19 positivity during hospitalization			
	Total (*n*)	OR (95% CI)	*χ*2 statics	*p* value	Total (n)	OR (95% CI)	*χ*2 statics	*p* value
Hospital-acquired pneumonia	43	2.275 (1.154–4.485)	5.218	0.022	84	2.896 (1.820–4.608)	19.34	< 0.001
DVT	4	NA	NA	NA	8	2.085 (0.490–8.878)	0.916	0.338
PE	158	12.95 (8.795–19.06)	202.2	< 0.001	70	0.903 (0.507–1.608)	0.122	0.729
Mortality during hospitalization	16	0.693 (0.186–2.586)	0.137	0.5730	55	6.303 (3.440–11.55)	39.09	< 0.001

*P* values of < 0.001 are considered significant by the *χ*2 test

DVT means deep vein thrombosis; PE means pulmonary embolism; OR means Odds Ratio; CI means confidence interval

### Impact of COVID-19 positivity during hospitalization for patients with proximal femur fractures

There were 531 and 1,593 patients of COVID-19 positivity during hospitalization after one to three PS matching according to age, sex, and comorbidities. The characteristics are shown in Table 4. For each parameter, SMD was < 0.1. For a COVID-19-positive patient, the waiting days before surgery were significantly longer, and the ICU admission rate was significantly higher. The risk of developing hospital-acquired pneumonia and mortality during hospitalization was increased to 2.896 (95% CI: 1.820 − 4.608) and 6.303 (95% CI: 3.440 − 11.55), respectively (Table 5).

## Discussion

This large cohort study utilized DPC data to examine the impact of COVID-19 on elderly patients with proximal femur fractures. The findings indicate a significantly higher incidence of PE during the COVID-19 period. While the mortality rate was slightly higher during the COVID-19 period compared to before, and the incidence of hospital-acquired pneumonia was slightly lower, these differences were not statistically significant. Additionally, our study found that detecting COVID-19 positivity at admission increased the risk of PE, although the anticoagulant medication rates had no significant difference. COVID-19 positivity during hospitalization was also associated with higher rates of hospital-acquired pneumonia and increased mortality. This study is one of the few to compare national data on patients with proximal femoral fractures before and during the pandemic. Few studies have been conducted in Japan using large-scale data, and our previous research on proximal femoral fractures using DPC data, including this study, has been useful in understanding the overall medical care image [26–28].

As is well known, the pneumonia induced by COVID-19 causes severe acute respiratory syndrome. The findings from other studies consistently suggest the association of the pandemic with increased mortality for non-COVID-19 sepsis and pneumonia [29, 30]. Patients with proximal femur fractures who tested positive for COVID-19 had a higher mortality rate compared to those who tested negative [21–23, 31–36]. While COVID-19 positivity during hospitalization is associated with an increased risk of hospital-acquired pneumonia and mortality, COVID-19 positivity during hospital admission is not identified as a risk factor for these outcomes in our study. Although the incidence of hospital-acquired pneumonia appeared lower in the target group, the result did not reach our predefined significance threshold (*P* = 0.0102 vs. the cutoff of P < 0.001) and therefore was not considered statistically significant. Detection of COVID-19 at the time of hospitalization allows for early intervention, which could potentially lead to timely and more effective treatment outcomes.

Proximal femur fractures undergoing surgical treatment from 2019 to 2020, preoperative characteristics, and 30-day postoperative complications differed significantly in 2020 compared to 2019, the incidence of PE and DVT was higher in 2020 compared to 2019 [37]. COVID-19 has been reported as a significant risk factor for DVT and PE. The incidence rate ratios within 30 days after a COVID-19 diagnosis are strikingly high, with a rate of 5.90 for DVT and an even more alarming 31.59 for PE, when compared to the control period. This indicates a substantial increase in the risk of these conditions following a COVID-19 infection, underscoring the severe implications of the virus on cardiovascular health [38]; the risk of DVT and PE among COVID-19 patients in Japan was reported to be lower compared to other countries, limited to severe COVID-19 [39–41]. Several studies suggest that COVID-19 induces in situ pulmonary thrombosis via endothelial injury and inflammation, rather than embolism from peripheral DVT [42, 43]. COVID-19 may cause pulmonary thrombosis through mechanisms different from classical DVT to PE pathways. One study indicated that while severe COVID-19 patients faced a significant risk of thrombosis, especially PE, the occurrence of DVT was relatively low[44]. Among ICU-admitted patients with COVID-19, both DVT and PE were commonly observed; however, PE posed a more significant clinical concern [45]. The incidence of PE also differs depending on the surgical procedure[26]. The study showed a significantly higher incidence of PE in patients with COVID-19 at the time of hospitalization; the coexistence of fractures and COVID-19 may increase the risk of PE from our data. On the other hand, patients with COVID-19 positivity during hospitalization had a reduced risk of PE, although this was not significant. The reason may be that anticoagulant therapy was administered after surgery and that many of the patients had stabilized fracture sites and were able to get out of bed.

Unexpectedly, the waiting days before surgery were significantly shorter during the COVID-19 pandemic or countermeasure periods. Despite the pressure of COVID-19 on the healthcare system, the time from hospital admission to surgery for proximal femur fractures did not increase. This may reflect the high prioritization of proximal femur fractures in Japan’s healthcare system, maintaining early surgical intervention even under pandemic pressures. However, the data show variability in the timeliness of surgical intervention: 48.1% of patients underwent surgery within 36 h of hospital arrival in 2018, 58.6% in 2019, and 44.9% in 2020. This indicates a decrease in early surgery during the first year of the pandemic, suggesting that while the overall time to surgery did not increase, the proportion of patients receiving timely surgery was affected [11]. COVID-19-positive patients, especially when detected at admission, were a factor in delaying surgery in the study. These findings are consistent with previous reports [19]. An observational study using the UK database found that geriatric, orthopedic, and obstetric evaluations tended to be delayed during the COVID-19 lockdown. Despite these delays, patients could be operated on earlier. This highlights the adaptive capacity of healthcare systems to maintain timely surgical intervention in the face of disruptions in other areas of patient care during a pandemic. This highlights the adaptive capacity of healthcare systems to maintain timely surgical interventions even when faced with disruptions in other areas of patient care during the pandemic [46]. A study showed that it is important to maintain a high level of multidisciplinary collaborative care even during the pandemic [47]. Patients who waited longer for surgery had more complications and a higher mortality rate [19]. In the study, delayed surgery was associated with in-hospital complications and mortality during hospitalization; action is needed to incentivize earlier surgery. On the other hand, patients with COVID-19 positivity at the time of hospitalization showed a lower risk of death, although not significantly. However, because patients with proximal femur fracture and severe COVID-19 were likely managed conservatively, the mortality rate for patients who were COVID-positive on admission is not accurate in our data.

The ICU stay has been reported to be associated with mortality as with the waiting period for surgery [48]. The study found that ICU admissions were not significant during the COVID-19 pandemic or countermeasure periods, although a particularly increased admission rate among those testing positive for COVID-19. A study demonstrated that during the COVID-19 pandemic, the duration of ICU stays for patients with geriatric proximal femur fractures was significantly shorter compared to the period before the pandemic. This reduction in ICU stay length was attributed to prioritizing ICU beds for COVID-19 patients, leading to a higher threshold for ICU admission for other conditions [48]. Although the duration of ICU stay was not measured in this study, there was an association between ICU admission and positive for COVID-19.

Despite predictions that proximal femur fractures might decrease during the COVID-19 pandemic because social distancing and restrictions on going out as a whole during the COVID-19 pandemic would limit outdoor activity in elderly patients and reduce the risk of falls, fragility fractures, including proximal femur fractures, have not decreased during the COVID-19 have not decreased during the pandemic [15, 19, 20]. In Japan, the rate of outdoor fractures decreased significantly after the emergency statement on COVID-19 was issued [17]. On the other hand, it is inferred that indoor fractures have not reduced proximal femur fractures during the COVID-19 period. In this study, osteoporosis treatment rates during the COVID-19 pandemic period were higher than before COVID-19, yet osteoporosis treatment rates remain low, even among hip fracture patients. Additional preventive measures are needed for proximal femur fractures in the elderly.

## Limitations

There are several limitations in the present study. First, the study population included patients with hip fractures treated only in acute-care hospitals reporting to the DPC data system. This did not include patients admitted to non-DPC reporting beds, which account for 30% of all general hospital beds or those who have never been treated in an acute care hospital [49]. Secondly, the temporal relationship between pneumonia onset and COVID-19 infection remains indeterminate due to the limitations of the DPC data, which does not specify whether pneumonia developed before or after a positive COVID-19 test result. This uncertainty impedes the ability to definitively ascertain whether pneumonia is a consequence of COVID-19 infection. In the case of severe COVID-19, the disease requiring the most medical resources is assigned to COVID-19. Since this study included surgical cases, the data may exclude patients with severe pneumonia induced by COVID-19. DPC data does not include laboratory data or image findings, it was difficult to accurately determine the severe grade, and thus standardization of diagnostic methods for COVID 19 could not be verified. Another limitation is that detailed treatment protocols were not available in the DPC dataset, and treatment heterogeneity may influence outcomes. Third, the analysis was restricted to surgical intervention cases, excluding those receiving conservative treatment. Patients involving severe COVID-19 combined with proximal femur fractures were likely managed conservatively, which suggests that the study's findings may not fully represent the outcomes for all patients affected by these conditions [23]. Fourth, we observed differences in PE and DVT prevalence rates in this study. It was considered that the diagnostic rate for DVT might be lower because evaluations for DVT were not conducted after the identification of PE by computed tomography scans intended for assessing pulmonary dysfunction.

In conclusion this nationwide medical claims database study compared patients undergoing hip fracture surgery before and during the COVID-19 pandemic and the response period. We found that waiting times for surgery were shorter, with no significant increase in mortality during hospitalization or the response period. The presence of COVID-19 at the time of hospitalization was associated with an increased risk of PE. In addition, COVID-19 positivity during hospitalization was associated with an increased incidence of hospital-acquired pneumonia and higher mortality rates in the study.

## Supplementary Information

Below is the link to the electronic supplementary material.

Supplementary file1 (DOCX 15 KB)

## Abbreviations

COVID-19: Coronavirus Disease 2019
CI: Confidence interval
DPC: Diagnosis procedure combination
PS: Propensity score
DVT: Deep vein thrombosis
PE: Pulmonary embolization
ICU: Intensive care unit
SMD: Standardized mean differences

## Declarations

This manuscript has not been published elsewhere and is not under consideration by another journal.
