# Supplementary material for: Increased complications of proximal femur fractures during the COVID-19 pandemic: a nationwide medical claims database study in Japan
Source: J Bone Miner Metab. 2025 Jun 10;43(5):493–503. doi: 10.1007/s00774-025-01611-0 (PMC12620312; doi:10.1007/s00774-025-01611-0)
Supplement: Supplementary file 1 — Supplementary file1 (DOCX 15 KB) [file 774_2025_1611_MOESM1_ESM.docx]

| ICD 10 code of Cormobidities and Outcome measures | |
| --- | --- |
| Comorbidities |  |
| Hypertension | I10, I110, I119, I120, I129, I139, I150, I151, I152, I159 |
| Dementia | F00, F00.0, F00.1, F01, F02, F02.0, F02.3, F03, G30 |
| Diabetes | E10, E11, E13, E14, E100, E101, E102, E103, E104, E105, E106, E107, E109, E110, E111, E112, E113, E114, E115, E116, E117, E119, E130, E131, E132, E133, E134, E135, E136, E137, E139, E140, E141, E142, E143, E144, E145, E146, E149 |
| Cerebrovascular disease | I600, I601, I602, I603, I604, I605, I606, I607, I608, I609, I610, I611, I613, I614, I615, I616, I618, I619, I620, I621, I629, I630, I631, I632, I633, I634, I635, I636, I638, I639, I64, I650, I651, I652, I653, I660, I661, I662, I663, I668, I669, I670, I672, I673, I674, I675, I676, I677, I678, I679, I690, I691, I693, I694 |
| Ischemic heart disease | I200, I201, I208, I209, I210, I211, I212, I213, I214, I219, I220, I221, I228, I229, I230, I231, I232, I233, I234, I235, I236, I238, I240, I241, I248, I249, I251, I252, M300, M301, M302, M303, M308 |
| Chronic renal dysfunction | N19, N181, N182, N183, N184, N185, N189 |
| Chronic lung disease | J47, J430, J431, J432, J439, J440, J441, J448, J449, J840, J841, J849 |
| Outcome measures |  |
| pneumonia | J13, J14, J150, J151, J152, J153, J154, J155, J156, J157, J158, J159, J160, J180, J181, J182, J188, J189, J202, J690, J958 |
| DVT | I800, I801, I802 |
| PE | I269 |
| DVT means deep vein thrombosis; PE means pulmonary embolism | |
